# Soft electroporation for delivering molecules into tightly adherent mammalian cells through 3D hollow nanoelectrodes

**DOI:** 10.1038/s41598-017-08886-y

**Published:** 2017-08-17

**Authors:** Valeria Caprettini, Andrea Cerea, Giovanni Melle, Laura Lovato, Rosario Capozza, Jian-An Huang, Francesco Tantussi, Michele Dipalo, Francesco De Angelis

**Affiliations:** 1 Istituto Italiano di Tecnologia, Genoa, 16163 Italy; 2 Università degli studi di Genova, Genoa, 16126 Italy

## Abstract

Electroporation of *in-vitro* cultured cells is widely used in biological and medical areas to deliver molecules of interest inside cells. Since very high electric fields are required to electroporate the plasma membrane, depending on the geometry of the electrodes the required voltages can be very high and often critical to cell viability. Furthermore, in traditional electroporation configuration based on planar electrodes there is no *a priori* certain feedback about which cell has been targeted and delivered and the addition of fluorophores may be needed to gain this information. In this study we present a nanofabricated platform able to perform intracellular delivery of membrane-impermeable molecules by opening transient nanopores into the lipid membrane of adherent cells with high spatial precision and with the application of low voltages (1.5–2 V). This result is obtained by exploiting the tight seal that the cells present with 3D fluidic hollow gold-coated nanostructures that act as nanochannels and nanoelectrodes at the same time. The final soft-electroporation platform provides an accessible approach for controlled and selective drug delivery on ordered arrangements of cells.

## Introduction

Gaining the access to the cell cytoplasm is one of the main tools for the biological research. Over the years, several methods to overcome the physical barrier of the cellular membrane have been developed with the aim to perform intracellular delivery^1^. The most common intracellular delivery methods are chemical-induced permeabilization^2^, viral vector transfection^3^, electroporation^4^, acoustic-transfection^5^ and laser induced opto-poration with or without the aid of plasmonic nanostructures^6–8^. Each of these methods has specific advantages and disadvantages in terms of efficiency, invasiveness and costs. For example, chemical induced cell poration is very efficient and quick for obtaining mass poration; however, it is extremely difficult to perform poration only to selected sub-ensembles of the cell culture or to control the timing of pores opening. The electroporation approach can be used to permeabilize the cellular membrane either in a transient or in a permanent way. This is possible by applying to the cells a train of electrical pulses that increases the transmembrane potential leading to the formation of nanopores. Those apertures can be exploited to gain access to the cytoplasm and deliver inside the cell foreign DNAs, drugs, fluorophores or other specific molecules. Electroporation has been traditionally performed by applying large voltages between flat electrodes separated from each other by some millimeters to reach high electric field in the cell medium solution.

Performing electroporation can lead to cell death if the parameters are not optimized for the system under investigation. In particular, electroporated cells can apparently reseal but slowly die in 24 h after the electrical train pulse is applied (long term death) if the permeabilized area results too spread over the cell membrane, or if the permeabilization lasts too long, allowing an imbalance in the metabolic pathways or in the homeostatic mechanisms^9^. Additionally, if the electric field applied over the all cell culture becomes too high or the time of the pulses too long, the nanopores exceed a critical radius and the cellular membrane is no longer able to reseal leading to the cell death^10^. This irreversible electroporation finds its use in the treatment of cancerous cells of *in vivo* studies^11, 12^, whereas the opening of transient hydrophilic pores into the cellular membrane is widely used in biology and medicine to perform transfection of cells, to develop genetic or cancer therapies and to study induced pathologies^13, 14^.

Recently, electroporation of adherent cells has also been obtained by exploiting three-dimensional (3D) nanostructures with sharp tips in the few nanometers range, which can concentrate the applied electric field on nanometer size tips where the cells are adhering. Consequently, the required potentials for electroporation are lowered from hundreds of volts to few volts^15^.

Cell membrane electroporation by means of hollow 3D nanostructures such as nanostraws or nanochannels has been shown to be a very effective drug delivery method^16, 17^. In these approaches two microfluidic compartments are separated by a membrane that includes pass-through nano-channels. By culturing cells on one side of the membrane and by applying electrical pulse trains between the two fluidic compartments, cells are electroporated and molecules can be delivered into the adherent cells. Since the electroporation works due to current flowing through the electrolyte within the nano-channels, there is an electrophoretic effect on the charged molecules that are easily driven inside the cells^18, 19^. To generate the required electric field to electroporate the cells within the nanochannels, high or very high potential differences are applied to the systems. However, such platforms have some drawbacks. For example, in one case^16^ the fabrication technique to obtain the three-dimensional nano-channels does not allow defining precisely their placement, size and shape; this feature makes it hard to define with nanometer precision the regions of cells that are planned to be targeted for delivery. Moreover, these configurations might allow molecules to access the cytoplasm not only through the nano-channels that is from the underlying microfluidic chamber, but also from the cell culture. In fact, to our knowledge, it has not been shown in literature that nano-channels electroporation avoids intracellular delivery of molecules present in the cell culture media.

The required potential difference applied to obtain reliable electroporation with configurations used in other works is between 6 V and 20 V (Xie *et al*.)^16^ and as high as 140 V (Chang *et al*.)^17^. Although no biocompatibility issues have been shown to arise from the applied voltages, these potentials are well above the potential needed to generate electrolysis of water molecules (~1.23 V)^20^ meaning that, during the applied pulses, Reactive Oxygen Species (ROS) may be created. ROS can be harmful for cell metabolism inducing oxidative stress, starting processes of apoptosis and mutating DNA and RNA^21^. Further lowering the required potential to porate the plasma membrane below the electrolysis threshold would be beneficial for the healthiness of the culture and the preserving of the genetic code of the cells under investigation. Theoretical studies already explored the minimum potential difference required to open nanopores on the plasma membrane, providing an evaluation of approximately 1V^18^.

**Figure 1:**
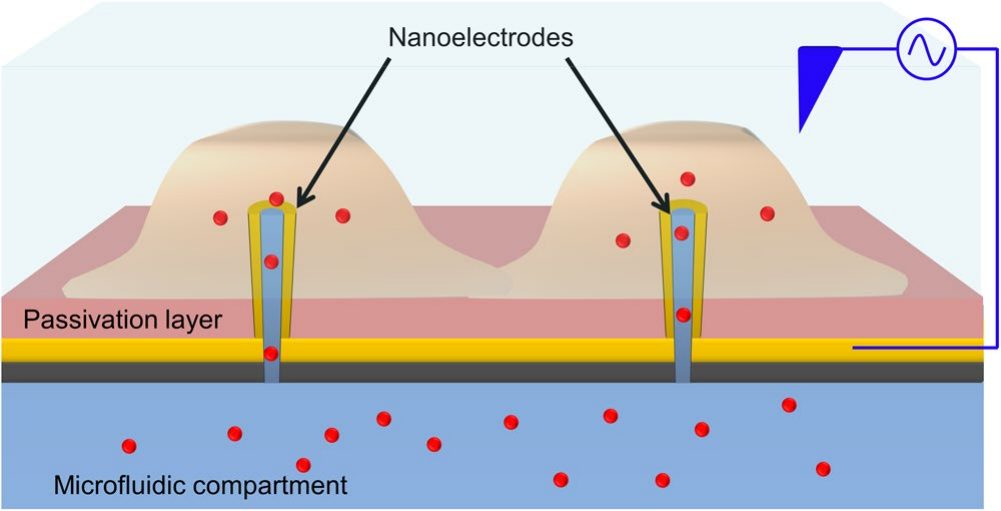
Sketch of the system. 3D hollow nanoelectrodes are protruding outside of a SU8 passivation layer and are connected to a separated microfluidic chamber. The cells adhere on the nanoelectrodes and molecules can flow through the nanochannels after the electrical field is applied.

In this work we present a device able to reliably perform cell membrane electroporation at very low-voltage (<2 V) and to deliver molecules into well-defined and *a priori* selected groups of cells. As shown in the sketch in Fig. 1, the method is based on 3D gold hollow nanostructures protruding from an insulating substrate that defines two separated fluidic compartments. Thanks to the lightning rod effect, high electric fields are generated at the tip of the metallic nanostructures, as simulated by a previous study^22^; the tip is in tight contact with the cellular membrane, leading to cell poration only in correspondence to the nanofluidic channel with very low voltages. Such a soft-electroporation does not affect the membrane adhesion on the insulating substrate and does not compromise the cell viability. This approach improves the above-mentioned methods by exploiting the electrical and nanofluidic properties of gold coated 3D hollow nanocylinders. These metallic nanoelectrodes are directly in contact with the cellular membrane that grabs them tightly, providing a tight sealing that avoids dispersion of the delivered molecules to the rest of the culture.

## Experimental Section

**Figure 2:**
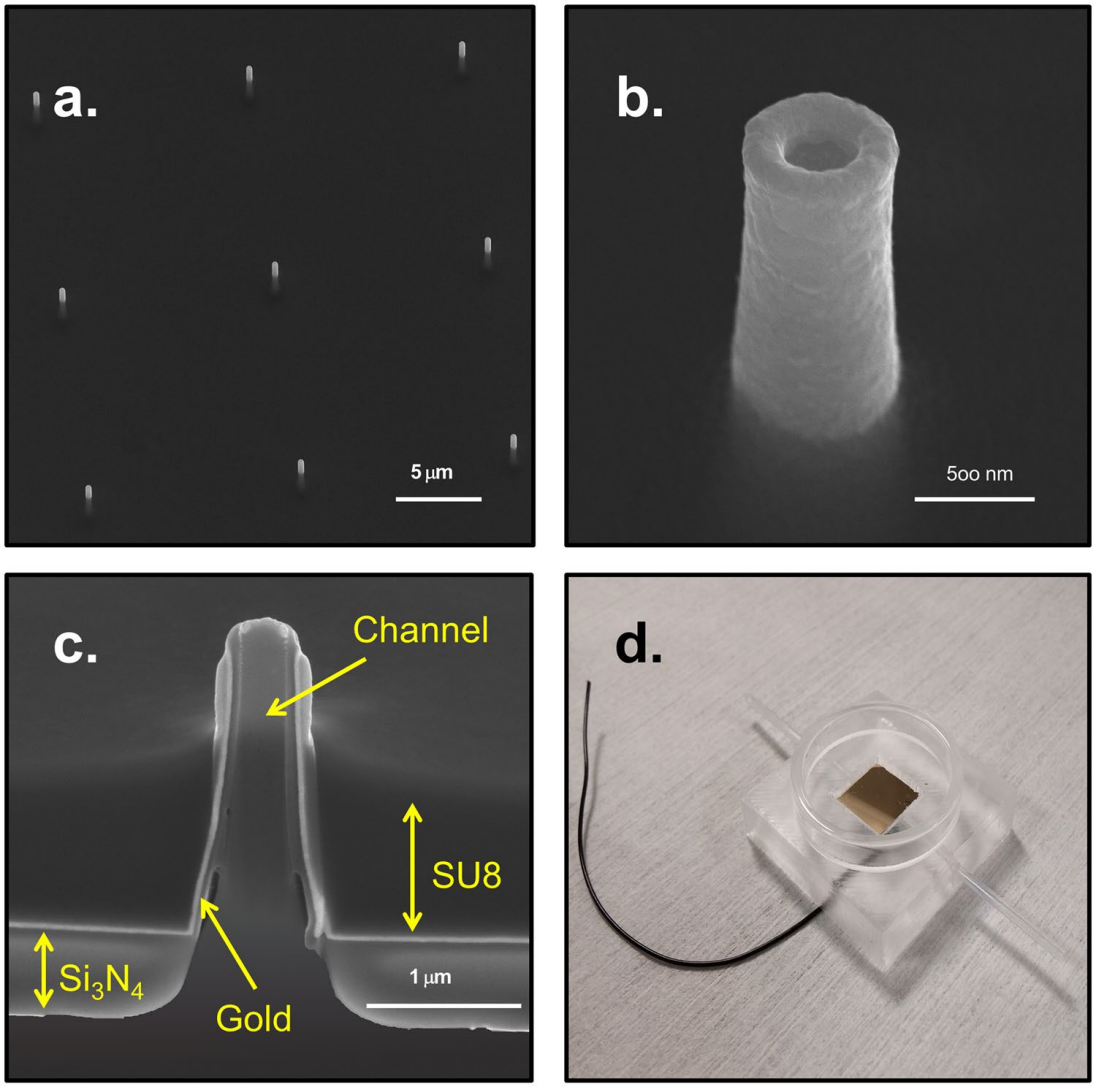
(**a**–**c**) SEM images of the 3D hollow nanoelectrodes embedded in the epoxy polymer SU8. Respectively, 3 × 3 array of nanoelectrodes, single nanoelectrode and cross section of a single hollow nanoelectrode. The different layers are indicated by the yellow arrows. (**d**) Top view of the PDMS microfluidic chamber. The wire is coming out from the PDMS being bonded by silver paste with the gold spattered onto the device making all the nanoelectrodes connected together. The glass ring around the device allows the cell culture on to the nanofluidic electrodes.

The proposed microfluidic platform is based on a nanofabrication technique discussed in a previous study^23^. Shortly, this technique involves the milling by mean of Focused Ionic Beam (FIB) of a thin Si_3_N_4_ membrane spin-coated with an optical resist (S1813). This very recent technique offers several advantages in respect to established approaches for the fabrication of hollow 3D nanostructures. It offers full and precise control over geometry and arrangement of the nanostructures and in addition, provides very fast fabrication rates; those two features are usually complementary and are rarely combined in a single technique. The resulting nanostructures are ordered 3D hollow nanochannels made of inverted resist (see Fig. 2a); these hollow nanochannels represent nanofluidic passages between the two sides of the nitride membrane.

To obtain biocompatibility and electrical conductivity, the nanochannels and the nitride membrane are coated with gold; this system has already been exploited to perform laser plasmonic opto-poration of cell membrane and intracellular delivery^24^ or intracellular recording on cultured cells^25^. The final 3D hollow nanostructures are 1,8 μm high and are coated with 60 nm of gold; the inner nanochannels have a diameter of 250 nm, while the external diameter is around 400 nm. The top of the resulting hollow nanocylinders has a toroidal shape with a sharp edge of approximately 80 nm. At this nanometer scale the potential applied during electroporation generates high electric fields exactly in the place where the cells present the strongest adhesion.

We used gold as coating of the 3D nanoelectrodes for two main reasons: first, it is biocompatible and a well-established substrate for *in-vitro* cell growth^26, 27^. Secondly, gold represents a suitable compromise between electrochemical properties, chemical stability, manufacturability and cost-effectiveness. It is expected that similar results may be obtained by using other materials, such as platinum or iridium oxide; however, gold is a suitable candidate to perform electroporation on cultured cells with low voltages and high spatial resolution.

The last fabrication step is the complete passivation of the gold planar surface of the device with a dielectric material; in addition to the planar surface, the thickness of this passivation is set so as to cover the base of the 3D hollow nanostructures and to leave exposed only their nanometer tips. The nanometer edge of the hollow nanoelectrode (Fig. 2b) is where the electric charge concentrates because of the tip effect, thus generating a local intense electrical field even when a low-voltage is applied. Moreover, such passivation strategy ensures that the entire planar surface is electrically insulating and does not contribute to apply any potential difference at the cell membrane. In fact, irregularity in the metal deposition on the flat substrate can concentrate charges leading to random membrane poration far from the hollow nanoelectrodes. Consequently, this passivation allows membrane poration to happen only where the cells are in direct contact with the nanostructures tips, whereas the rest of the cells membrane remains in tight adhesion with the passivation material. Therefore, any molecule flowing through the nanochannels can be intracellularly delivered only to the attached cell, because the molecule movement is completely limited by the sealing of the cell membrane on the passivation surface. Similarly, molecules that are present in the cell medium cannot pass through the nanopores in the porated membrane because they cannot overcome the passivation/membrane tight barrier. This behavior is analogous to that observed in a previous work^24^ in which the poration was obtained by laser excitation only of 3D nanostructures while the rest of the cell membrane remained intact and adherent.

We investigated different approaches to establish the most suitable biocompatible and stable passivation. The method that led to the best result is the spin-coating of a layer of the epoxy polymer SU8 onto the membrane with gold-coated nanochannels. The SU8 layer is spin-coated with a thickness that roughly matches that of the 3D nanoelectrodes; subsequently, a plasma etching with high power and low concentration of oxygen reduces the SU8 thickness to the point that only approximately 700 nm of the gold nanoelectrode protrude from the flat substrate (Fig. 2a,b and c). The inversion of the SU8 and the subsequent hard-baking make the resist chemically inert, stable and biocompatible.

The 3D nanoelectrodes are electrically connected together by the gold layer deposited on the flat surface of the nitride membrane and beneath the SU8. This configuration creates a single large electrode that is wired to an external pulse generator.

The 3D hollow nanochannels communicate with the other side of the Si_3_N_4_ membrane allowing the flow of molecules from an isolated area, on the back of the device in which no cells are cultured, to the cellular culture. We exploited this feature of our device to deliver molecules only to the porated cells and exclusively through the nanochannels. In order to reach this goal, we developed a microfluidic chamber (Fig. 2d) in which the cells are grown on the 3D nanoelectrodes while the molecules are kept separated from the biological culture in a microfluidic channel beneath the nitride membrane.

The microfluidic chamber is made in PDMS and a glass ring is bonded on it to create the well in which the cells are cultured. A wire is attached to the flat gold substrate with silver paste and embedded into the PDMS to be connected to the pulse generator.

Electroporation was performed applying a pulse train between the 3D hollow nanoelectrodes and a platinum electrode immersed in the electrolyte (PBS). The overall system (nanoelectrodes, cell membrane, and electrolyte) had a resistance ranging from 6 to tens of MΩ.

## Results and Discussion

The surface of our devices consists of cured SU8 with protruding gold vertical nanoelectrodes. Cells have been shown to adhere strongly on SU8 and on 3D gold nanostructures^28^, therefore cells cultured on our microfluidic platform adhere tightly on both the planar surface and on the tip of the 3D hollow nanoelectrodes. Being the cell membrane in tight adhesion with the gold nanoelectrode tips, even with a pulse voltage train as low as 1.5 V applied between the electrodes, the cell will feel an intense electrical field. A rough calculation of the effective electric field generated on the tip of the electrode can be found in the Supplementary Information (section 2). This electric field is exploited to temporarily permeabilize the cellular membrane creating small holes in correspondence of the nanoelectrode tips. In this way, the nanochannels work both as electrodes as well as nanofluidic channels, enabling molecules to diffuse from the underlying microfluidic chamber into the cytoplasm of porated cells without affecting the untargeted ones.

**Figure 3:**
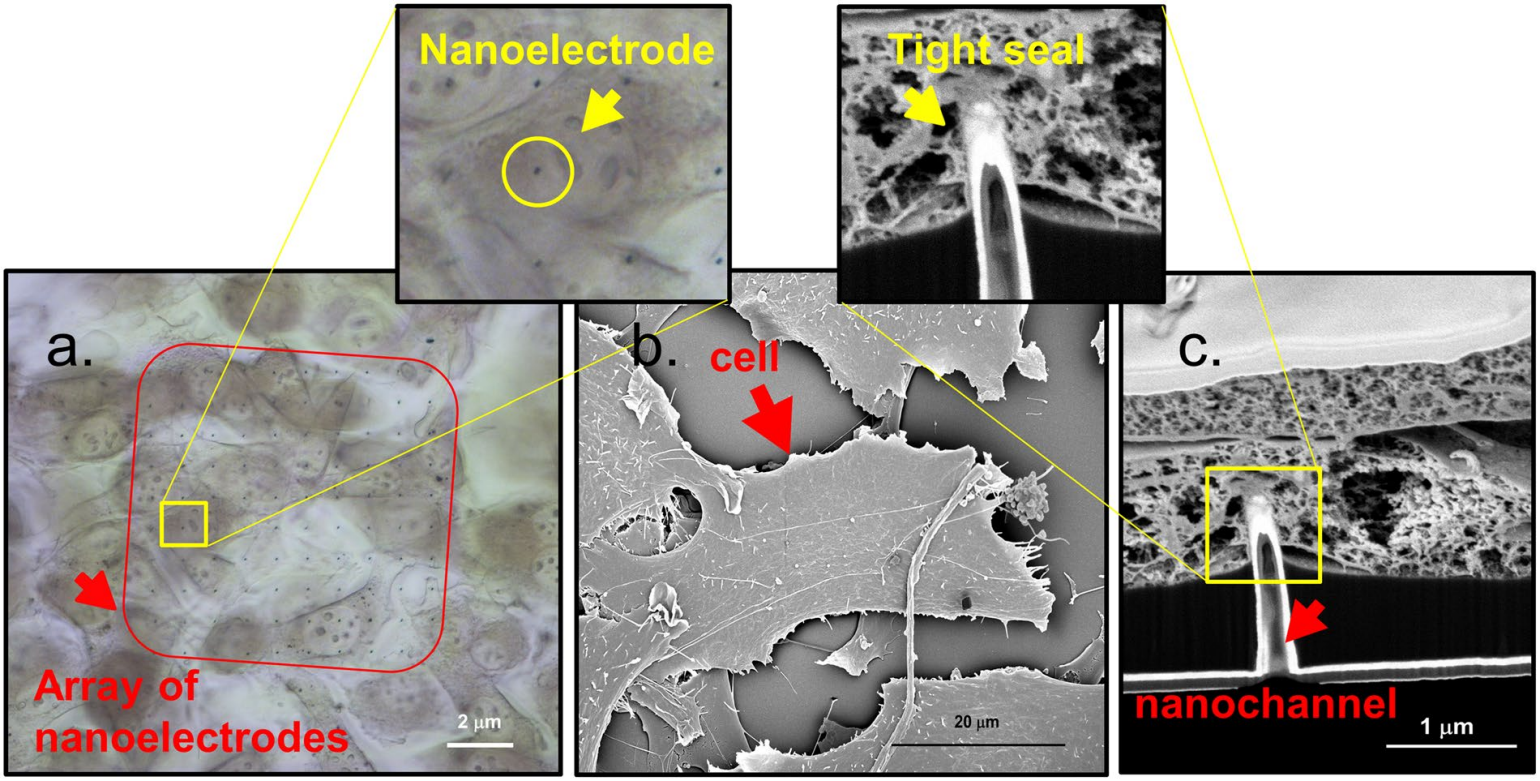
(**a**) Transmission image of NIH-3T3 cells fixed on an array of hollow nanoelectrodes – the black dots inside the red square – embedded in SU8. (**b**) SEM top view image of CPD dried NIH-3T3 cells cultured on top of the hollow nanoelectrodes. (**c**) Cross section of CPD dried cell grew on top of a hollow nanoelectrode. From this image it is possible to see all the layers of the fabrication (Si_3_N_4_ membrane 100 μm thick, SU8) and the hollow 3D nanoelectrode that is engulfed by the cellular membrane.

As already showed in several works^16, 24^, the sharpness of the 3D nanostructure promotes a tight adhesion between the cell and the electrode, preventing the fluxed molecules to diffuse in the cellular medium and allowing the diffusion only inside the porated cell. In fact, growing in adhesion with the hollow nanoelectrodes, the cells completely engulf the 3D nanostructures ensuring the lipid membrane to be in direct contact with the metal (Fig. 3a,b and c). Moreover, the low height of the nanoelectrodes (~700 nm) protruding from the flat SU8 passivation layer does not prevent the attachment and the proliferation of the cellular culture on the SU8 planar surface^29^.

**Figure 4:**
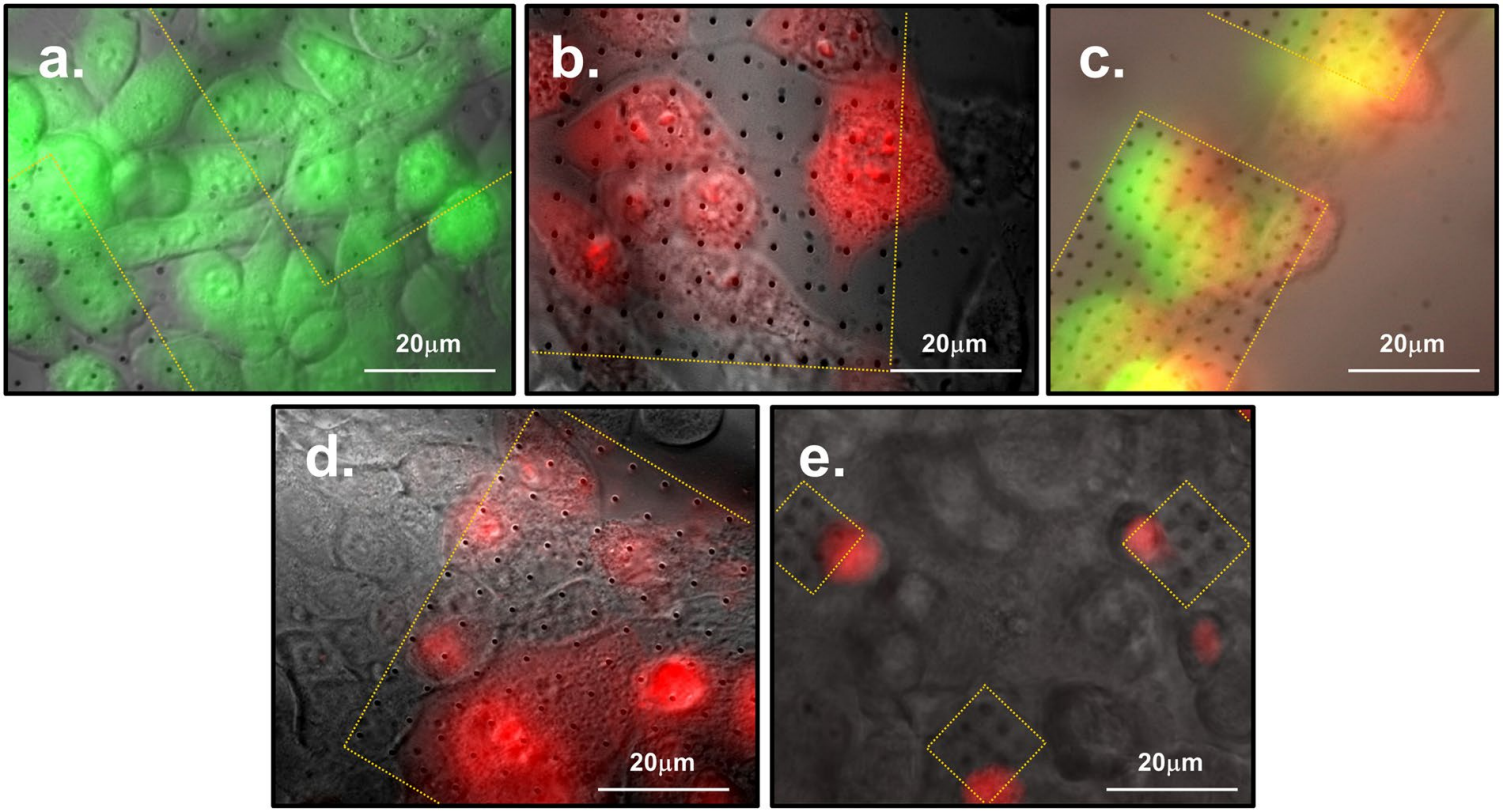
Images of electroporated cells by hollow nanoelectrodes and delivered with PI (red) and Calcein AM (green). Only the cells that lie on top of the 3D nanoelectrodes/nanochannels (inside the dotted yellow areas) are delivered after the soft-electroporation protocol, while the rest of the population remains unstained. (**a**) NIH-3T3 cells incubated with Calcein AM (green) in a live dead essay. (**b**) NIH-3T3 cells soft-electroporated and delivered with impermeable dye P.I. (red) through the nanofluidic electrodes. (**c**) NIH-3T3 cells stained both with Calcein AM and with PI after the soft-electroporation. The yellow color in the image is an artifact due to the double staining, green and red. (**d**) Cardiac muscle cells HL-1 electroporated with the soft-electroporation protocol and stained by P.I. through the hollow nanoelectrodes. (**e**) HL-1 cardiomyocytes electroporated and delivered with P.I. through small patterns (10 μm by 10 μm) of 3D hollow nanoelectrodes. Approximately only one cell per array has been stained, confirming the tight sealing between the plasma membrane and the flat SU8 passivation. This new design paves the way towards single cell access.

NIH-3T3 cells were stained both with a permeable dye, Calcein AM, in the culture well to perform a vitality test (Fig. 4a,c) and with the membrane-impermeable dye Propidium Iodide (PI) flowing through the nanochannels from the bottom compartment (Fig. 4b,c). As it is shown in Fig. 4a, the presence of the nanoelectrodes does not influence the healthiness of the culture; in fact, both the cells that lie on top of the nanoelectrodes and those that grew on the flat SU8 substrate are stained by the Calcein AM. In Fig. 4b PI is delivered into the NIH-3T3 cells through the nanochannels by applying the soft electroporation protocol. Figure 4c shows NIH-3T3 cells stained with both Calcein AM in the culture well and Propidium Iodide through the nanochannels after the soft electroporation; the results are cells with double staining (yellowish) as expected.

With the idea to create a platform that can be exploited with several types of cells, we tested our system and the soft-electroporation parameters also on hard-to-deliver cells, as the cardiac muscle cells line HL-1. The cardiomyocytes were grown *in vitro* until confluency with a coating on the devices of poly-L-lysine (PLL) as adhesion factor^15, 25^. As it is shown in Fig. 4d and e, we obtained the poration and delivery of molecule dyes inside the cardiac muscle cells only on the cells that lie on the 3D patterned surface. We also fabricated very small 3D hollow nanoelectrode arrays (10 μm by 10 μm, dimensions comparable with a single cardiomyocyte cell) with a nanoelectrode pitch of 5 μm and a distance between patterns of 50 μm. The intention was to get access to a single cell with each array to explore ways towards a single cell delivery system. We tested the new configuration with cardiomyocytes and indeed we managed to delivery PI almost only one cell with each array, as is it shown in Fig. 4e. The result is significant and gives an additional proof of the tight sealing between the SU8 flat passivation around the 3D metallic nanochannel and the plasma membrane.

To have a further prove of the stability of the tight seal, we performed experiments in which the Propidium Iodide was added directly in the cell culture media before the electroporation. The dye initially stained few already dead cells however, when the pulse train was applied, no additional cell showed fluorescence, meaning that the tight seal between the cell membrane and the SU8 passivation did not allow the dye to reach the nanopores created on the 3D nanoelectrode tips. Our result also confirms that no additional cells died because of the soft-electroporation protocol (see Fig. S1).

**Figure 5:**
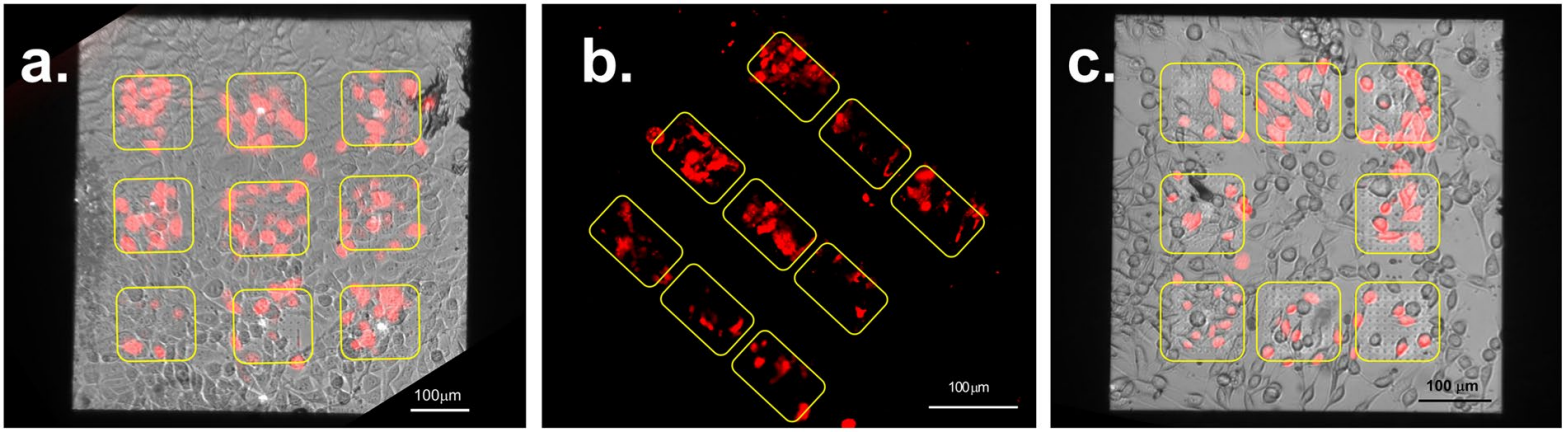
(**a**–**c**) Different spatial arrangement of the 3D hollow nanoelectrodes arrays framed by the yellow squares. Only the cells grew in adhesion with the hollow nanostructures are delivered with PI (in red) after the soft-electroporation. Cells in the images are NIH-3T3 and the scale bar is 100 μm for each image.

In Fig. 5a,b and c), different spatial arrangements of nanoelectrode arrays are patterned on the microfluidic device. When the soft electroporation protocol is applied, only the cells that lie on top of the nanoelectrodes are delivered through the nanochannels and become fluorescent. These results highlight the power of the presented delivery method; in fact, in our system we can decide the position, the spatial arrangement and the distance between the nanoelectrodes, creating predefined areas on the device on which cells are electroporated and delivered.

Our experiments show that over the 80% of the cells that are lying on top of the nanoelectrode arrays are porated and delivered with a 10 seconds pulse train of 2 V in amplitude and a frequency of 20 Hz being each pulse 100 μs in length; the total amount of delivered pulses is then 200. The cell viability after the poration is >98%. Throughout our electroporation experiments, we observed that statistically the membrane poration starts to occur with voltages of approximately 1.5 V; however, a suitable poration success rate of 80% with a standard deviation of 7% is achieved with a voltage of 2 V. These results are calculated over 6 experiments with NIH-3T3 cells counting the number of cells lying on the hollow nanoelectrodes that have been porated in respect to those who remained unstained even if in tight contact with the 3D nanostructures. These electroporation parameters are comparable with previous results in which metallic nanopillars have been used for cell membrane electroporation^15^, although in this particular work the electroporation was exploited for intracellular recording of action potentials rather than for drug delivery; those nanostructures were indeed bulk without an inner nano-channel. By considering other works in which electroporation has been used for drug delivery by means of 3D hollow nanochannels^16^, we notice that, in our case, electroporation can be reliably performed with considerable lower voltages. Such low applied voltage avoids the formation of free ROS in the cell bath, being comparable with the water molecules electrolysis threshold and therefore helping the long term healthiness of the culture. The generation of a strong enough electrical fields to open transient nanopores on the plasma membrane applying a very low voltage it is due to the proximity of the cell to the metallic nanoelectrodes that, in our fabrication, coincide with the nanochannel and produce a lightning rod effect.

One additional advantage in having a very low voltage applied is that the metal coating of the 3D nanoelectrodes will not suffer degradation due to water molecules electrolysis, making the device recyclable after proper cleaning. Electroporation experiments have been done also with higher electrical pulse amplitudes. Above 4 V, we observed the formation of microscopic electrolytic gas bubbles that are generated from the tips of the nanoelectrodes; these bubbles disappeared shortly after the end of the electrical pulse train. Amplitude between 2 and 4 V were used successfully to porate and delivery cells without indications of cell damage.

## Conclusion

In this work we presented an optimized intracellular delivery method based on ordered arrays of 3D metallic nanostructures interfaced with a microfluidic chamber. The fabrication method exploited in this work allowed us to produce ordered patterns of hollow nanoelectrodes and consequently to porate specific group of cells among a larger culture. Thanks to the tight adhesion of the cellular membrane on the 3D hollow nanoelectrodes, the system can electroporate adherent cultured cells with low voltage and to selectively deliver molecules only inside the porated cells. The novelty of this fabrication relies on the fact that the nanoelectrodes used for electroporation are simultaneously metallic, hollow and communicate through its nanochannels with an isolated microfluidic chamber beneath the device. The voltage required to open transient pores in the cellular membrane is lower than others previously reported for the intracellular delivery of molecules; most importantly, the required voltage is comparable with the water electrolysis threshold avoiding the formation of reactive oxygen species in the cell culture medium. In fact, a suitable and reproducible cell electroporation is obtained with the application of voltages as low as 2 V both for standard cell lines and hard-to-transfect cells such as cardiomyocytes. In such conditions, no negative side effects are observed on the cell viability and the 3D hollow nanoelectrodes do not suffer degradation due to the water electrolysis, making the device recyclable. Moreover, lowering the potential difference to electroporate the cells paves the way toward the combination of these 3D nanofluidic electrodes with voltage-sensible recording systems such as Multi Electrode Arrays (MEAs)^25^.

## Methods

### Fabrication

The 3D hollow nanoelectrodes are milled in a Si_3_N_4_ membrane of different thickness depending on the area (larger membranes are made from thicker silicon nitride in order to maintain a suitable robustness). Before milling, the membranes are spin coated with S1813 from Shipley. To create the 3D nanostructures a Helios Nanolab 650 dual beam from FEI was used with different ion currents to create nanochannels of different sizes (i = 80pA for a nanochannel with inner diameter of 100 nm, i = 0,79 nA for nanochannels with an inner diameter of 250 nm). After the exposure to the ions the S1813 is dissolved into acetone and only the 3D nanostructures made of inverted resist remain on the membrane. The nanochannels and all the membrane are covered by sputter coating technique with 30 nm of gold.

### Passivation

Epoxy polymer SU8 2000.5 diluted 1:2 in the SU8 thinner both by MicroChem Corp. (Newton, MA, USA) onto the Si_3_N_4_ membrane with gold-coated nanochannels to have the height that matches the one of the 3D nanoelectrodes. The resist is then pre-baked at 95 °C for 3 minutes, it is exposed to the UV light with a dose of 80mJ/cm^2^, post-baked for 2 minutes at 95 °C and developed in the SU8 developer, MicroChem Corp. (Newton, MA, USA). A plasma etching of the SU8 was performed at 200 W with 30% of O_2_ for 90 seconds to reduce its height. Hard baking is performed for 1 hour at 200 °C.

### Microfluidic Chamber

The microfluidic chamber is made in PDMS Sylgard 184 elastomer by Dow Corning (Midland, MI, USA). Its mold was printed in ABS polymer with a 3D printer CubePro. The PDMS is degassed before the curing that occurs for 2 hours at 65 °C in a hoven. The silver paste used to connect the device to the wire is purchased by RS Component Ltd.

### Cell culture and electroporation

To perform electroporation and delivery experiments we used NIH-3T3 cells and cardiac muscle cells HL-1. Before the seeding of the cells, the devices have been treated with 60 seconds of plasma oxygen (100% O_2_, 100 W) and then sterilized with 20 minutes UV exposure in laminar flow hood. The NIH-3T3 cells were seeded on the microfluidic devices at a concentration of 1.5 × 10^4^ cells/cm^2^ and incubated at fixed temperature and CO_2_ concentration (37 °C, 5%) for 36 h before performing the experiments into DMEM with 1% of Pen/Strep antibiotic and 10% of Fetal Bovine Serum (FBS) (Sigma Aldrich). Before the seeding of the cardiac muscle cells line HL-1, the devices were treated 5 minutes with 0.01% poly-L-lysine solution (Sigma Aldrich) in order to increase the cell adhesion, washed extensively with distilled water and dried in sterile conditions. After the functionalization step, 8.5 × 10^4^ cells/cm^2^ were seeded and grown with Claycomb culture medium (Sigma-Aldrich) supplemented with 10% FBS, 100 μm Norepinephrine, 300 μM ascorbic acid, 2mM L-glutamine and 100 μg/ml penicillin/streptomycin (Sigma-Aldrich). Cells were grown in incubator at 37° and 5% CO_2_ concentration, with a daily change of culture medium until 100% confluence was reached.

Electroporation was performed applying a pulse train of 2 V, 100μs, 20 Hz (standard soft-electroporation protocol) for 10 seconds between the 3D hollow nanoelectrodes and a platinum electrode immersed in the electrolyte (PBS, Sigma Aldrich).

### Fixation and imaging of cells

The cells are fixed with gluta 2,5% in 0,1 M of sodium cacodylate, both by Sigma Aldrich, for 2 hours on ice and then stained with 1% osmium tetroxide by EMS (2 h) and 1% of Uranyl Acetate (4 h). After the staining the cells are dehydrated in Ethanol (30%, 50%, 70%, 90%, 96%, 100%) by Sigma Aldrich and dried by Critical Point Dryer (CPD) technique. Before the imaging the sample is coated with 5 nm of gold by sputter coating technique. The SEM images are performed with a FEI Helios Nanolab 650 dual beam, as well as the cross sections. Roughly 600 nm of Pt is deposited with the Gas Injection System (GIS) of the dual beam before the cross section of the cells.

### Microscopy

The microscope used for the fluorescence images is an upright Ecplise FN-1 from Nikon. The fluorescence images are taken with 10x long working distance objective and with a 60x water immersion objective by mean of the fluorescent camera Hamamatsu Orca Flash R2. The Calcein AM and the Propidium Iodide, both Sigma Aldrich, are excited respectively by a Nikon FITC and a Nikon TRITC.

## Electronic supplementary material

Suppplementary Information
