# Supplementary material for: Soft electroporation for delivering molecules into tightly adherent mammalian cells through 3D hollow nanoelectrodes
Source: Sci Rep. 2017 Aug 17;7:8524. doi: 10.1038/s41598-017-08886-y (PMC5561120; doi:10.1038/s41598-017-08886-y)
Supplement: Supplementary file 1 — Suppplementary Information [file 41598_2017_8886_MOESM1_ESM.pdf]

Supplementary Information

Soft electroporation for delivering molecules into tightly adherent mammalian cells through 3D hollow nanoelectrodes

Valeria Caprettini, Andrea Cerea, Giovanni Melle, Laura Lovato, Rosario Capozza, Jian-An Huang, Francesco Tantussi, Michele Dipalo, Francesco De Angelis

S.I. 1: Tight sealing between the 3D nanoelectrodes and the cells.

In order to verify the tight sealing between 3D nanoelectrodes and cells, we added Propidium Iodide (PI) in the cell culture bath and we compared the number of stained cells before and after the application of the soft-electroporation protocol.

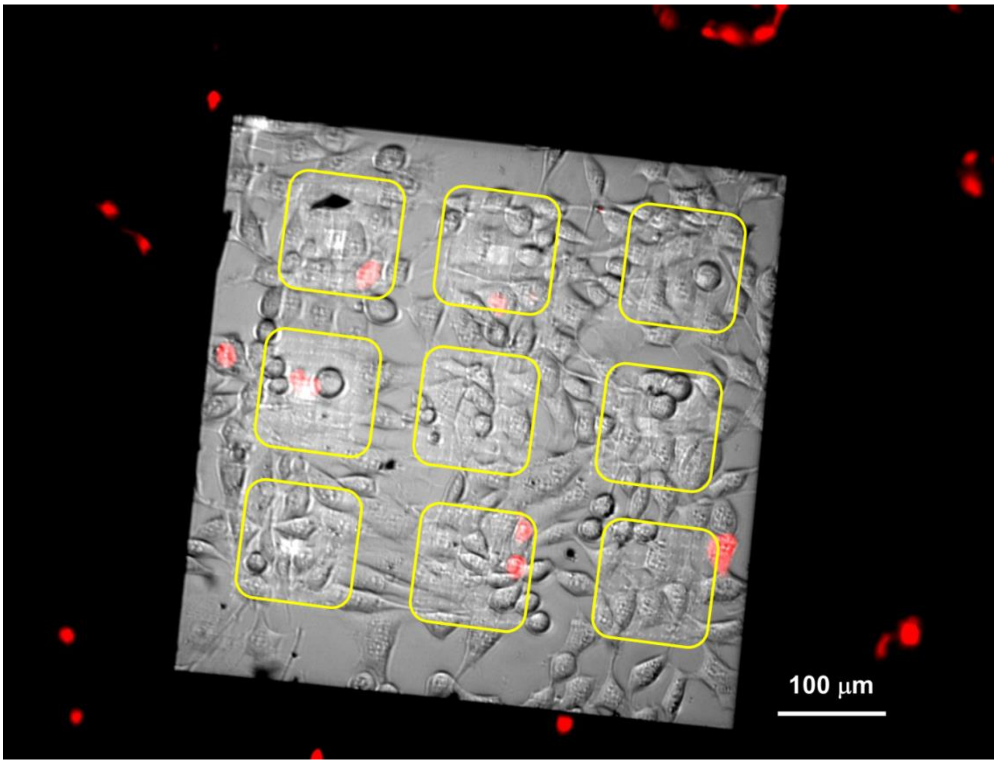

Figure S1. Fluorescence images after the soft-electroporation protocol of a cell culture in which Propidium Iodide has been added to the cell medium. The yellow squares represent the hollow nanoelectrode arrays.

As it is shown in Figure S1, only a small amount of cells are stained by the Propidium Iodide, and the average density of stained cells is comparable on the Si_3N_4 membrane (the light grey area) and on to the full cell culture. The number of the stained cells do not change after the pulse train is applied, meaning that the nanopores in the cell membrane are formed only at the

interface with the hollow nanoelectrodes. The percentage of stained cells on the 3D hollow nanoelectrode is approximately 4% of the total amount of cells in adhesion on the membrane. With this experiment we can state that the sealing between the cell membrane and the planar SU8 passivation is tight. Soft-electroporation opens nanopores only in adhesion with the 3D nanostructure, preventing molecules from the cellular culture to penetrate into the cytoplasm.

S.I. 2: Electric field between a uniformly charged sphere and an infinite plate

We have estimated the strength of electric field in the proximity of the tip, where we assume the cell in contact with the nanoelectrode, by a simple analytical model. We model one electrode (the tip of the nanochannel) as a uniformly charged sphere with a radius *a*=200 nm while the second electrode is represented by an infinitely large plate at distance *d*=10mm (see Fig. S2).

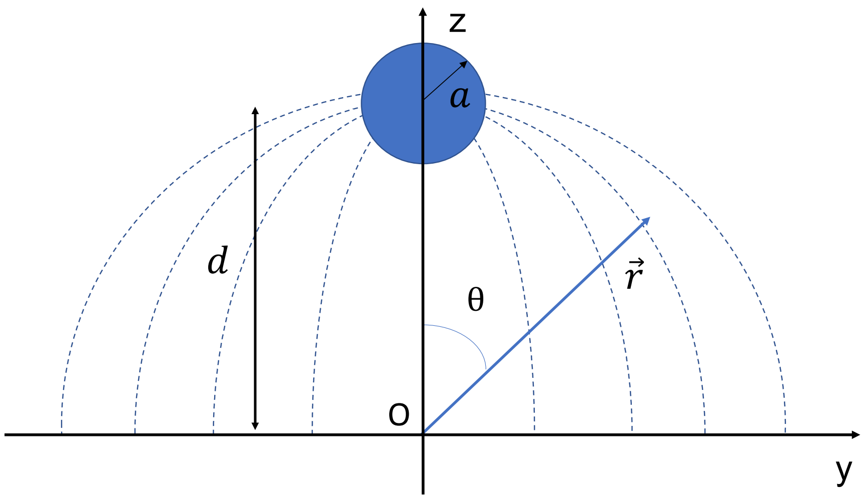

Figure S2. Scheme of the analytical model under discussion. One electrode is represented by the infinitive large plate along the y-axis, while the second electrode, the nanometric tip of the 3D hollow nanoelectrode, is represented by the sphere with radius a.

By employing the method of images and superposition principle, the potential V can be calculated (in spherical coordinates) as:

$$V(\vec{r}) = \frac{V_0}{\left(\frac{1}{a} - \frac{1}{2d}\right)} \left(\frac{1}{\sqrt{d^2 + r^2 - 2rd\cos\theta}} - \frac{1}{\sqrt{d^2 + r^2 + 2rd\cos\theta}} \right)$$

where V_0 is the voltage between electrodes, r the radial coordinate and θ the angle between $\vec{r}$ and the vertical axis in a reference frame with origin on the infinite plate and vertical axis passing through the center of the sphere.

The electric field can be calculated as $\vec{E}(\vec{r}) = -\vec{\nabla}V(\vec{r})$. On the segment connecting the origin with the center of the sphere $\theta = 0$:

$$\vec{E}(r, \theta = 0) = \frac{V_0}{\left(\frac{1}{a} - \frac{1}{2d}\right)}\left(\frac{1}{(d+r)^2} - \frac{1}{(d-r)^2}\right)$$

Considering that d >> a and that the applied voltage between the electrodes V_0 = 2V, a straightforward calculation gives a value of the electric field of $\vec{E}(d - a, \theta = 0) \approx 10^7$V/m on the surface of the sphere. This calculation shows that employing the field enhancement at the tip of the antenna we can achieve strong electric fields with relatively small voltages.
